# Supplementary material for: Acceptance and compliance with micronutrient powder (MNP) among children aged 6–23 months in northern Nigeria
Source: PLOS Glob Public Health. 2022 Oct 17;2(10):e0000961. doi: 10.1371/journal.pgph.0000961 (PMC10022258; doi:10.1371/journal.pgph.0000961)
Supplement: S1 Dataset — (ZIP) [file pgph.0000961.s008.zip › S8_Dataset/Direct Observations.zip!/Direct Observations/180605_A_SO_AD_1_DO.pdf]

1.1.1 Instrument F –Direct Observation Form

<<Instrument F Direct Observation Form>>
**PI Name:** PragyaMathema,Stephen Kodish<br>**Study Title:** Formative research to develop an infant and young child feeding (IYCF) intervention with micronutrient powder (MNP) for children aged 6 – 23 months<br>**PI Version/Date:** Version1.0/January 24, 2015

Date: _June 18, 2015____________Location: __Bongo community, Song LGA_________

Data Collector: Aisha Dahiru______________Participant ID: _________________________

Main goals of this direct observation are to answer the following questions:
1. In this household, what would be the best way(s) to give nutritional supplements to young children? Why?<br><br>2. In this household, describe the food sharing that you observe throughout the meal. Are there any predictable patterns?<br><br>3. In this household, who is favored and how is this favoritism expressed during meal preparation and eating?

(TEAR OFF AT PERFORATED DOTTED LINE BELOW)

PARTICIPANT ID: _________

Household Composition Table (Fill out as you do data collection)

INITIALS	SEX (M/F)	AGE (estimated)	Relationship to household	Description
W	F	26 YRS	Mother	Fair complexion. Wearing a white shirt with a purple head gear.
H	M	35 YRS	Father	Dark complexion. Wearing blue trouser and a black shirt.
G	F	17 YEARS	Mothers sister	Dark skinned has facial mark and wearing green wrapper.
B	M	10 MONTHS	Baby boy	Wearing a sweater with brown trousers.
M 1	M	32 YRS	Fathers brother	Wearing yellow shirt and a blue jeans.
M 2	M	30 YRS	Fathers brother	Wearing green shirt and a black jeans

S	M	8 yrs	Older son	Wearing green checked shirt with green trouser

PARTICIPANT ID: _________

TIME	ACTOR INITIALS	RECIPIENT INITIALS	WHAT HAPPENED	FOOD	Reactivity
8.00	W		sweeping		
8:05	W		sweeping		
8:10	W		Sweeping		
8:15	W		Sweeping		
8:20	W		sweeping		
8:25	W		sweeping		
8:30	B		Crying		
8:35	W	B	Backing baby		
8:40	W		Cooking	Kunu gyada (ground nut	

TIME	ACTOR INITIALS	RECIPIENT INITIALS	WHAT HAPPENED	FOOD	Reactivity
				gruel)	
8:45	W		Cooking	Kunu gyada (ground nut gruel)	
8:50	W		Cooking	Kunu gyada (ground nut gruel)	
8:55	W		Washing of dishes		
9:00	W		Washing of dishes		
9:05	W		Washing of dishes		
9:10	W		Washing of dishes		
9:15	W	B	Bathing the baby		
9:20	W	B	Bathing the baby		
9:25	W	B	Dressing the baby		
9:30	H	B	Playing with the baby		
9:35	H	B	Carrying the baby		
9:40	W		Adding MNP to the child food	Kunu gyada (ground nut gruel)	
9:41	W		Mixing the MNP in the food	Kunu gyada (ground nut gruel)	
9:42	W	B	Feeding the baby the portion of kunu that was mixed with MNP	Kunu gyada	

TIME	ACTOR INITIALS	RECIPIENT INITIALS	WHAT HAPPENED	FOOD	Reactivity
				(ground nut gruel)	
9:43	W	B	Feeding the baby	Kunu gyada (ground nut gruel	
9:44	W	B	Feeding the baby the remaining portion without MNP	Kunu gyada (ground nut gruel	
9:45	W	B	Feeding the baby		
9:46	W	B	Feeding the baby		
9:47	W	B	Washing the baby's hands and mouth		
9:48	W	B	Backing the baby		
9:53	W	B	Backing the baby		
9:58	W	B	Backing the baby		
10:03	W	B	Lying the baby to sleep		
10:08	B		Sleeping		
10:13	B		Sleeping		
10:18	B		Sleeping		
10:23	W		Stepped outside		
10:28	B		Sleeping		
10:33	B		Sleeping		
10:38	G		Making up		

TIME	ACTOR INITIALS	RECIPIENT INITIALS	WHAT HAPPENED	FOOD	Reactivity
10:43	G		Still wearing make up		
10:48	G		Pouring kunu from flask	Kunu gyada (ground nut gruel	
10:53	W		Checking on the baby		
10:58	B		Sleeping		
11:03	B		Sleeping		
11:08	W		Pouring kunu into cup	Kunu gyada (ground nut gruel)	
11:13	W		Drinking kunu	Kunu gyada (ground nut gruel)	
11:18	W		Drinking kunu	Kunu gyada (ground nut gruel)	
11:23	G		Drinking kunu	Kunu gyada (ground nut gruel)	
11:28	G		Drinking kunu	Kunu gyada (ground nut gruel)	
11:33	B		Sleeping		

TIME	ACTOR INITIALS	RECIPIENT INITIALS	WHAT HAPPENED	FOOD	Reactivity
11:38	W		Sitting		
11:43	B		Sleeping		
11:48	B		Sleeping		
11:53	H		Came in holding firewood and cutlass		
11:58	H		Washing hands and feet		
12:03	W		Lying down		
12:08	W		Lying down		
12:13	B		Sleeping		
12:18	W		Lying down		
12:23	W		Checking on the baby		
12:28	B		Wakes up crying		
12:33	W		Carrying baby		
12:38	W		Changes the baby's cloth		
12:43	W		Holding the baby		
12:48	H		Playing with the baby		
12:53	B		Crawling on the ground		
12:58	M1		Walk into the house		
12:58	M2		Walk into the house		
12:58	M2	B	Playing with baby		
01:03	B		Playing		
01:08	B		Playing		

TIME	ACTOR INITIALS	RECIPIENT INITIALS	WHAT HAPPENED	FOOD	Reactivity
01:13	H		Pouring water from jerry cans into clay pots and plastic containers		
01:18	B		Watches father pour water		
01:23	B		Playing		
01:28	W		Playing with baby		
01:33	H		Discussing		
01:38	W		Picks something from the kitchen and hands to husband		
01:43	B		crying		
01:43	W	B	Picks baby up and holding him		
01:48	W	B	Rubbing oil on baby's head		
01:53	W	B	Breastfeeding baby		
01:54	W	B	Breastfeeding baby		
01:55	W	B	Breastfeeding baby		
02:00	W	B	Playing with the baby		
02:05	B		Playing		
02:10	B		Playing		
02:15	B		Playing		
02:20	B		Playing		
02:25	B		Playing		
02:30	B		Playing		
02:35	W		Peeling orange	Orange	

TIME	ACTOR INITIALS	RECIPIENT INITIALS	WHAT HAPPENED	FOOD	Reactivity
02:40	W		Peeling orange	Orange	
02:45	H	M1, M2	Carrying empty jerry cans outside. Discussing		
02:50	B		Playing		
02:55	B		Playing		
03:00	B		Playing		
03:05	W	B	Giving baby orange	Orange	
03:06	H	G	Gives sister orange	Orange	
03:07	G	B	Feeds baby with orange	Orange	
03:08	B		Eating orange	Orange	
03:09	B		Eating orange	Orange	
03:10	B		EATING ORANGE	Orange	
03:15	W		Setting fire		
03:20	W		Setting fire		
03:25	W		Setting fire		
03:30	W		Pick basket from kitchen		
z					
03:35	W		Peeling onions	Onions	
03:40	W		Pick and rinse pot		
03:41	W		Pours oil into pot	Oil	
03:42	W		Put onion into the pot	Onions	
03:43	W		Adds dried fish into the pot	Fish	
03:45	W		Place the pot on the fire		
03:50	W		Stirs the content of the pot	Oil, onion ,	

TIME	ACTOR INITIALS	RECIPIENT INITIALS	WHAT HAPPENED	FOOD	Reactivity
				fish	
03:52	W		Washes pepper	Pepper	
03:53	W		Puts pepper into a mortar	pepper	
03:53	W		Pounding pepper	Pepper	
03:54	W		Pounding pepper	Pepper	
03:55	W		Pounding pepper	Pepper	
04:00	W		Adds the pepper into the pot	pepper	
04:01	W		Stirring the food		
04:05	W		Adds water into the pot	Water	
04:06	W		Covers the pot		
04:07	G	B	Playing		
04:08	G	B	Playing		
04:09	W		Playing with the baby		
04:10	W		Playing with the baby		
04:15	W		Folding clothes		
04:20	W		Taking empty containers into the kitchen		
04:25	W		Check he food on the fire		
04:30	W		Adds other ingredients into the food	Salt, maggi	
04:30	H		Carrying jerry cans into the house		
04:35	H		Pouring water from jerry cans into plastic containers		
04:40	W		Emptying water from the akamu that was kept since morning	Akamu	
04:45	W		Adding fresh clean water into the akamu	Akamu	
04:50	G	B	Playing the baby		
04:55	B		Playing		
05:00	G	B	Steps outside with the baby		
05:01	B		Playing under the tree with other children		
05:01	W		Checking the food on the fire		
05:02	W		Adds baobab flour to the pot and stirs	Baobab flour	
05:03	W		Stirs the soup	Baobab soup	
05:05	W		Puts the pot down from fire		
05:06	W		Places another pot on the fire with little water in it	Water	
05:06	W		Puts the tuwo to warm it up	Tuwon	

TIME	ACTOR INITIALS	RECIPIENT INITIALS	WHAT HAPPENED	FOOD	Reactivity
				masara (maize meal)	
05:10	W		Bring down the pot with tuwo		
05:11	W		Serves the food	Tuwon masara (maize meal) and baobab soup	
05:12	W		Washing the baby's hands		
05:12	G		Washing the hands		
05:13	W		Feeding the baby from her bowl	Tuwon masara (maize meal) and baobab soup	
05:14	W		Feeding the baby from same bowl	Tuwon masara (maize meal) and baobab soup	R
05:15	W		Eating meal together with sister	Tuwon masara (maize meal) and baobab soup	
05:15	G		Eating meal together with sister	Tuwon masara (maize meal) and baobab soup	

TIME	ACTOR INITIALS	RECIPIENT INITIALS	WHAT HAPPENED	FOOD	Reactivity
05:16	W	B	Feeding the baby from same bowl	Tuwon masara (maize meal) and baobab soup	R
05:17	B		Eating with his hands from the others bowl	Tuwon masara (maize meal) and baobab soup	
05:18	H	B	Feeding the baby from same bowl	Tuwon masara and baobab soup	R
05:19	H		Eating meal together with sister	Tuwon masara (maize meal) and baobab soup	
05:20	H		Feeding the baby	Tuwon masara (maize meal) and baobab soup	
05:20	H		Giving the baby water to drink	Water	
05:21	H		Washing the baby's hands and mouth		
05:21	H		Washing hands		
05:22	G		Washing hands		

TIME	ACTOR INITIALS	RECIPIENT INITIALS	WHAT HAPPENED	FOOD	Reactivity
05:23	G		Washing the dishes		
05:25	G		Drying the dishes		
05:30	H		Placing baby on potty		
05:35	B		Sitting the baby on potty		
05:40	H	B	Washing the baby		
05:41	H	B	Bathing the baby		
05:46	H	B	Dressing the baby		
05:51	H	B	Backing the baby		
05:56	H	B	Backing the baby		
06:01	H	B	Backing the baby		
06:01	S		Walks into the house wearing a uniform		
06:06	S		Changes cloths		
06:10	S		Washing of hands		
06:15	W	S	Serves the older son food	Tuwon masara (maize meal) baobab soup	
06:20	S		Eating his meal	Tuwon masara (maize meal) baobab soup	
06:25	S		Washing hands after meal		
06:30	S		Drinking water from clay pots	Water	

TIME	ACTOR INITIALS	RECIPIENT INITIALS	WHAT HAPPENED	FOOD	Reactivity
06:35	S	B	Playing with the younger son		

PARTICIPANT ID: _________

Primary Questions (Complete after you complete data collection):

From what you have seen observing THIS HOUSEHOLD, please answer the following questions in detail:

Question	Write your detailed response here
1. In this household, what would be the best way(s) to give nutritional supplements such as an MNP to young children to ensure compliance?<br><br>Why?	The best way to ensure compliance is to educate the mother on the importance of the MNP
2. In this household, who is favored during meal preparation or feeding?<br><br>How is this favoritism expressed?	The food prepared in the evening was solid and the younger child did not have a separate bowl.
3. How could we promote the MNP supplement for this family that would attract their attention?<br><br>Who would they listen to?	MNP can be promoted to this family through the mother .<br>The father ca also serve as an influence because he is in charge of decisions in the household .<br>The religious leaders can also play a vital role in the promotion of MNP

4. In this household, what would be the best ways to promote the MNP but limit how much it would be shared?<br><br>Why?	The best way is to ensure the mother understands that MNP is meant for children under 2 years and should be given to the child separately.<br>Sharing can also be avoided from the sequence of eating i.e. the baby eat first before the mother.
5. What other issues and concerns did you see with this family that are important for us to consider with regard to introduction of an MNP?<br><br>Why?	The mother was in charge of feeding the baby at some point and the mother's sister was also feeding the baby at other times.
6. Based on your experience observing this child today, do you believe reactivity negatively impacted your ability to see the food-related	Reactivity did not have negative impact on child's behavior . the MNP was administered properly as instructed and the mother fed the child herself.

behaviors of the child?<br><br>Please explain your answer in detail.	
